# Assessment of Adolescents’ Overweight and Obesity Risk Factors Among Alabnaa Schools in Tabuk City, Saudi Arabia

**DOI:** 10.7759/cureus.61533

**Published:** 2024-06-02

**Authors:** Rofayda Mansour Ahmed Mohamad, Waad Mohammed Alhawiti, Waheed Ali Alshehri, Rami Mahmood Alhaj Ali, Shatha Talal Alhakami, Manal Muhsen Alatawi, Abeer Ahmed Almutairi, Eman Saeed Al Atawi, Dalia Ghaleb Alkhaibari, Rakan Mahmoud Saleh, Hosam Hadi Awaji

**Affiliations:** 1 Preventive Medicine Department, King Salman Armed Forces Hospital, Tabuk, SAU

**Keywords:** risk factors, overweight, obesity, childhood, adolescents

## Abstract

Background: The prevalence of overweight and obesity among adolescents in Saudi Arabia has been progressively increasing. Obesity is associated with an increased risk of various morbidities and mortality. Identifying the factors that contribute to obesity in this age group is crucial for implementing targeted prevention measures.

Aim: The aim of this study was to identify risk factors for overweight and obesity among adolescents aged nine to 17 years residing in Tabuk City, Saudi Arabia.

Methods: A case-control study was conducted during the 2021-2022 academic year at Alabnaa Schools in Tabuk City, Saudi Arabia. The study included overweight/obese individuals (cases, n = 125) and normal-weight individuals (controls, n = 201) who were selected based on their body mass index and classified according to the World Health Organization's reference for defining overweight and obesity in individuals aged five to 19 years. Data were collected from both groups using a self-administered questionnaire.

Results: The study analyzed 125 overweight/obese students and 201 normal-weight students who were matched for sex and age (p > 0.05). Logistic regression analysis identified several risk factors for overweight or obesity among adolescents. A family history of obesity was found to be associated with a 5.735 times increased likelihood of obesity (95% CI: 3.318-9.912, p < 0.001). Another significant contributing risk factor for obesity was frequent consumption of four or more meals per day (adjusted odds ratio: 3.091, 95% CI: 1.094-8.736, p = 0.033). Students who used electronic devices for more than five hours were 2.422 times more likely to exhibit obesity (p = 0.006).

Conclusions: Certain factors may increase the risk of overweight or obesity in adolescents aged nine to 17 years. These factors include frequent eating, prolonged use of electronic devices, family history of obesity, and the misconception that obesity is not an illness. Tailored school health programs are needed to improve students' healthy lifestyles and eating behaviors, minimize sedentary entertainment and use of electronic devices, and engage children in physical activity.

## Introduction

Obesity is a global health problem affecting all age groups. According to a report from 2016, there were over 340 million children and adolescents suffering from overweight or obesity [1]. Additionally, being obese as a child or adolescent increases the likelihood of becoming an obese adult, which can have a negative impact on future population health [2]. Obesity is linked to an elevated risk of metabolic, cardiovascular, and gastrointestinal diseases, as well as psychological issues and premature death [3,4]. It also places a significant burden on the healthcare system.

Childhood obesity is a growing concern in developed countries and poses a significant threat to public health. In the United States, rates of obesity among children and adolescents have reached epidemic levels [5].

The prevalence rates of overweight and obesity in Saudi Arabia have been steadily increasing [6]. Al-Hussaini et al. [7] found an alarming increase in the prevalence of overweight and obesity among children and adolescents aged six to 16 years in Riyadh City. They attributed the rising rates of obesity to lifestyle changes such as sedentary behavior and poor dietary habits. Similar findings were found among school children aged six to 19 years in the western, central, and eastern regions of Saudi Arabia [8]. A systematic review also documented an increase in obesity among the Saudi population aged two to 20 years [9].

Identifying the factors that contribute to obesity in this age group is crucial for guiding health managers and other stakeholders in implementing targeted obesity prevention measures. This will help to reduce the burden of obesity and associated diseases [10]. Modifications to eating behaviors and lifestyle habits during childhood and adolescence are crucial. Schools are considered suitable settings that provide access to almost all children during a relevant developmental period [11].

Several studies have examined the potential risk factors for obesity among children and adolescents in Saudi Arabia [12-16]. However, limited information is available regarding the risk factors for adolescent obesity in Tabuk City, Saudi Arabia. Therefore, this study aimed to identify risk factors for overweight and obesity among adolescents aged nine to 17 years residing in Tabuk City, Saudi Arabia.

## Materials and methods

Ethical considerations

The Ethics Committee of King Salman Armed Forces Hospital in the north-western region of Tabuk, Saudi Arabia approved the study. Prior to participation, written informed consent was obtained from the school's manager (head teacher) and the students’ legal guardians. All data were coded and anonymously analyzed to guarantee the participants’ confidentiality.

Study design and setting

This case-control study was conducted at Alabnaa Schools, Tabuk City, Saudi Arabia during the academic year 2021-2022.

Sample size

The study's sample size was calculated to be 125 in the case group and 201 in the control group, assuming an odds ratio of 4 for the outcome in the exposed and unexposed groups.

Inclusion criteria

The case group consisted of overweight/obese students of both genders aged between nine and 17 years. A control group of normal-weight students matched for age and sex was also included.

Exclusion criteria

Students who were not between the ages of nine and 17 years or did not attend Alabnaa Schools were excluded from the study. Additionally, students with a history of endocrine diseases that may cause obesity, such as Cushing's disease or hypothyroidism, those on prolonged cortisone therapy, and those with special needs were also excluded.

Data collection

The research team selected the two groups based on the students' height, weight, and body mass index (BMI) using a convenience sampling method. The BMI (kg/m2) is an indirect measure of body fat and a simple diagnostic test for identifying childhood obesity [17]. Participants' weight and height were measured at school by a trained team of study investigators. Weight was measured to the nearest 100 g using an electronic scale with the children wearing light clothing and no shoes. Height was measured using a wall-mounted stadiometer with the children wearing no shoes. Measurements were recorded to the nearest 0.1 cm. The BMI was calculated as weight in kilograms divided by height in meters squared. According to the World Health Organization's (WHO) reference for defining overweight and obesity in those aged five to 19 years [18], overweight is defined as a BMI ≥ 1SD and obesity as a BMI ≥ 2SD of the median for age and sex. The case group consisted of overweight/obese students, while the control group consisted of normal-weight students. Data were collected from both groups through a questionnaire.

Research instrument

After obtaining ethical approval, we collected participants' data through a self-administered questionnaire. The questionnaire was designed based on a review of a previously published research article on obesity among adolescents [19] and was translated into Arabic. Two public health consultants reviewed it to confirm the clarity of the questions and content validity. The construct validity was evaluated by distributing the questionnaire to a pilot sample of students (n = 30) who were not included in the study. During the pilot test, participants' feedback on the comprehensibility and relevance of the questions was collected and analyzed. Based on this feedback, the questionnaire was refined to improve clarity. The questionnaire's reliability was then assessed in terms of internal consistency, inter-observer agreement, and intra-observer agreement. The survey consisted of two parts. The first part collected personal data, including age, sex, and class. The second part of the survey included questions about lifestyle factors, such as the number of meals consumed per day, the type of meals eaten at school, consumption of sugar-sweetened and carbonated beverages, physical activity levels, daily use of electronic devices, preferred leisure activities, hours of sleep per day, family history of obesity, and any attempts to lose weight.

Statistical analysis

IBM SPSS Statistics for Windows, version 27 (IBM Corp., Armonk, NY) was used to tabulate and analyze the data. Categorical data were presented as frequencies and percentages and compared between the cases and control groups using Pearson’s chi-square test. Numerical data were presented as mean and standard deviation and were compared using the independent T-test due to their normal distribution. Candidate variables that showed significant associations with overweight and obesity in the univariate analyses (p < 0.05) were included in a forward stepwise logistic regression analysis to develop a risk factor model. The results were presented as adjusted odds ratios and their corresponding 95% confidence intervals. A p-value < 0.05 was considered statistically significant.

## Results

The present study included a total of 326 participants, with 125 (38.3%) overweight/obese students as the case group and 201 (61.7%) normal-weight students as the control group. Both groups had a mean age of 16.5 ± 0.5 and comparable sex distribution with no significant difference (p > 0.05) (Table 1).

**Table 1:** Demographic characteristics of the studied groups. The data have been represented as N (%) or mean ± standard deviation (SD).

Variables		Overweight/obese (N = 125)	Control (N = 201)	P-value
Gender, n (%)	Male	34 (10.4%)	66 (20.2%)	0.283
	Female	91 (27.9%)	135 (41.4%)	
Age (year), mean ± SD		16.5 ± 0.5	16.5 ± 0.5	0.619

The overweight/obese students had a significantly higher frequency of eating four or more meals per day compared to the control group (p = 0.004). The overweight/obese students reported higher frequencies of consuming junk foods and sugar-sweetened beverages compared to normal-weight students, but these differences were not statistically significant (p = 0.217 and 0.191, respectively). However, the case group had a significantly higher consumption of carbonated beverages (79, 63.2%) compared to the control group (95, 47.3%) (p = 0.005). The students in the case group reported a significantly higher frequency of consuming more than two cans per day (p = 0.025). Both groups reported comparable levels of regular physical activity (p = 0.098), but the type of physical activity differed significantly (<0.001*). Overweight/obese students reported a lower frequency of high-effort sports, such as football, compared to normal-weight students (14 (16.9%) and 49 (50%), respectively). The physical activity locations significantly differed between the two groups (p < 0.001). Overweight/obese students primarily engage in physical activity at home (47, 54.7%), while normal-weight students mainly participate in sports at general gyms/clubs (61, 50.8%) (Table 2).

**Table 2:** Comparison between the studied groups regarding characters of meals, drinks, and physical activity. The data have been represented as N (%). * Significant at p < 0.05.

Variables		Overweight/obese (N = 125)	Control (N = 201)	P-value
		N (%)	N (%)	
Number of meals per day	˂2	58 (46.4%)	78 (38.8%)	0.004*
	2-3	52 (41.6%)	115 (57.2%)	
	4-5	14 (11.2%)	7 (3.5%)	
	>5	1 (0.8%)	1 (0.5%)	
Type of meal eaten in school	Junk food	28 (22.4%)	28 (13.9%)	0.217
	Normal food	4 (3.2%)	9 (4.5%)	
	Preserved food	72 (57.6%)	121 (60.2%)	
	All types	21 (16.8%)	43 (21.4%)	
Drinking sugar-sweetened beverages	Yes	83 (66.9%)	120 (59.7%)	0.191
	No	41 (33.1%)	81 (40.3%)	
Drinking carbonated beverages	Yes	79 (63.2%)	95 (47.3%)	0.005*
	No	46 (36.8%)	106 (52.7%)	
Number of carbonated beverages per day	One	48 (61.5%)	59 (66.3%)	0.025*
	Two	13 (16.7%)	23 (25.8%)	
	More than two	17 (21.8%)	7 (7.9%)	
Regular physical activity	Yes	86 (68.8%)	120 (59.7%)	0.098
	No	39 (31.2%)	81 (40.3%)	
Type of physical activity	Football	14 (16.9%)	59 (50.0%)	<0.001*
	Walking	49 (59.0%)	57 (48.3%)	
	Cycling	2 (2.4%)	1 (0.8%)	
	Tennis table	2 (2.4%)	0 (0.0%)	
	Others	16 (19.3%)	1 (0.8%)	
Duration of physical activity per week	1 hour	39 (45.9%)	79 (65.8%)	0.005*
	2-3 hours	24 (28.2%)	27 (22.5%)	
	4-5 hours	6 (7.1%)	8 (6.7%)	
	>5 hours	16 (18.8%)	<6 (5.0%)	
Place of physical activity	Home	47 (54.7%)	30 (25.0%)	<0.001*
	General gyms/clubs	15 (17.4%)	61 (50.8%)	
	Open place	24 (27.9%)	29 (24.2%)	
Advises about physical activities (for those not practicing it)	Yes, usually	8 (20.5%)	4 (4.9%)	0.067
	Yes, but not usually	12 (30.8%)	32 (39.5%)	
	Rarely	9 (23.1%)	21 (25.9%)	
	No	10 (25.6%)	24 (29.6%)	

Regarding the use of electronic devices, the types and purposes of daily used devices were similar in both groups. Additionally, both groups reported using devices as their preferred method of entertainment and that they eat while using the device (all p-values > 0.05). However, a significantly higher percentage of overweight/obese students reported using devices for more than seven hours per day compared to the control group (29 (23.2%) and 17 (8.5%), respectively, p = 0.002). Only 80 (68.4%) of the students in the overweight/obese group consider obesity to be an illness, compared to 171 (85.5%) in the control group, with a significant difference (p < 0.001). Additionally, significantly more participants in the overweight/obese group (75, 60.0%) reported a positive family history of obesity compared to those in the control group (46, 22.9%) (p < 0.001). Furthermore, the overweight/obese group exhibited a significantly greater desire and made more attempts to lose weight compared to the control group (p = 0.024 and <0.001, respectively) (Table 3).

**Table 3:** Comparison between the studied groups regarding using electronic devices and perceptions about obesity. The data have been represented as N (%). * Significant at p < 0.05.

Variables		Overweight/obese (N = 125)	Control (N = 201)	P-value
		N (%)	N (%)	
Types of daily devices used	Playing device	35 (28.0%)	79 (39.3%)	0.052
	Mobile	77 (61.6%)	113 (56.2%)	
	iPad	7 (5.6%)	6 (3.0%)	
	Laptop	6 (4.8%)	3 (1.5%)	
Duration of using the device per day, hours	2 or less	43 (34.4%)	83 (41.3%)	0.002*
	3-5	42 (33.6%)	86 (42.8%)	
	6-7	11 (8.8%)	14 (7.0%)	
	>7	29 (23.2%)	17 (8.5%)	
Uses of the device	Social communication	62 (49.6%)	92 (45.8%)	0.516
	News	15 (12.0%)	32 (15.9%)	
	Educational	27 (21.6%)	50 (24.9%)	
	Playing/watching films	20 (16.0%)	27 (13.4%)	
	Others	1 (0.8%)	0 (0.0%)	
Preferred ways of recreation	Physical activity	91 (72.8%)	142 (70.6%)	0.675
	Using device	34 (27.2%)	59 (29.4%)	
Eating while using the device	Yes	58 (46.4%)	85 (42.3%)	0.467
	No	67 (53.6%)	116 (57.7%)	
Sleeping hours per day	<8	46 (36.8%)	87 (43.3%)	0.247
	≥8	79 (63.2%)	114 (56.7%)	
Obesity is an illness	Yes	80 (64.00%)	171 (85.1%)	<0.001*
	No	37 (29.6%)	29 (14.4%)	
Family history of obesity	Yes	75 (60.0%)	46 (22.9%)	<0.001*
	No	50 (40.0%)	155 (77.1%)	
Desire to lose weight	Yes	110 (88.0%)	157 (78.1%)	0.024*
	No	15 (12.00%)	44 (21.9%)	
Trial of weight loss	Yes	87 (69.6%)	62 (30.8%)	<0.001*
	No	33 (26.4%)	128 (63.7%)	

Logistic regression analysis was conducted to identify significant risk factors for overweight or obesity among adolescents. The analysis showed that a family history of obesity was associated with a 5.735-fold increased likelihood of obesity (95% CI: 3.318-9.912, p < 0.001). Additionally, believing that obesity is not an illness was significantly associated with the development of obesity, with odds of 3.755 (95% CI: 1.952-7.226, p < 0.001). Frequent consumption of four or more meals per day was found to be a significant contributing risk factor for obesity (adjusted odds ratio: 3.091, 95% CI: 1.094-8.736, p = 0.033). Additionally, students who used electronic devices for more than five hours were 2.422 times more likely to exhibit obesity (p = 0.006) (Table 4).

**Table 4:** Multivariable logistic regression analysis (forward stepwise) for determining risk factors of overweight and obesity among adolescents. AUC: area under the curve; AOR: adjusted odds ratio; CI: confidence interval. * Significant at p < 0.05.

Predictors/risk factors	AOR	95% CI AOR	P-value	Accuracy	P-value model
Eating four or more meals per day	3.091	1.094-8.736	0.033*	73.8%	<0.001*
Using electronic devices for more than five hours	2.422	1.295-4.532	0.006*		
Family history of obesity	5.735	3.318-9.912	<0.001*		
Believing that obesity is not an illness	3.755	1.952-7.226	<0.001*		
Hosmer and Lemeshow test	0.904				
AUC (95% CI)	0.774 (0.720-0.829)				

## Discussion

Obesity is the fifth leading cause of death worldwide. The health consequences of obesity typically appear in adulthood, but the factors that contribute to the disease often originate in childhood [20]. To address childhood obesity and its negative health effects, it is crucial to examine the underlying factors associated with its prevalence. The aim of this study was to identify the risk factors for overweight and obesity in adolescents in Tabuk City, Saudi Arabia.

This survey conducted in schools documented modifiable lifestyle risk factors that contribute to obesity among adolescent students aged nine to 17 years. These factors include frequent consumption of four or more meals per day and prolonged use of electronic devices. Additionally, a family history of obesity and the misconception that obesity is not an illness were significant determining factors. These findings demonstrate the intricate interplay between genetic predisposition and environmental and lifestyle factors.

The study found that adolescents with a family history of obesity were over five times more likely to be obese. This is in line with a previous study in Saudi Arabia, which reported a 2.5-fold increased risk of obesity among adolescents with one or more obese family members [21]. A study conducted in Al-Ahsa, Saudi Arabia found a significant association between obesity among male adolescents and their parents' obesity [22]. This also agrees with Nurul et al. [23], who confirmed a five-fold increase in the risk of obesity among Malaysian adolescents with a family history of paternal obesity [23]. This association may be attributed to a genetic predisposition that makes family members more prone to high food intake, physical inactivity, and decreased metabolism, with a greater tendency to store body fat [24,25]. Furthermore, the risk of obesity among family members who share the same unhealthy dietary habits and physical inactivity is influenced by interactions between genetic variants and environmental factors [26].

Obesity is a chronic relapsing disease characterized by an inflammatory state and associated with several significant morbidities [27]. However, a significantly high percentage of overweight/obese adolescents in this study did not consider obesity an illness, which was a significant risk factor for obesity with 3.755 odds. According to Haghani et al. [28], educating school students about the causes and consequences of obesity, as well as modifying their lifestyle, can enhance their awareness and help control obesity.

The study found a relationship between dietary frequency and obesity. Eating four or more meals per day was associated with a more than three-fold increase in the risk of obesity. The role of eating frequency in the development of obesity is controversial and is related to the timing of eating and meal composition [29]. The significant contribution of frequent meals in this study is related to the imbalance between the consumed and the expended calories as the overweight and obese students preferred recreation through using devices and most of them practiced physical activity at home and their outdoor activities were mainly in the form of walking rather than football playing or cycling. A study of adults suggests that a high daily eating frequency associated with a healthy lifestyle and dietary pattern is associated with a reduced likelihood of general and central obesity in men [30].

The use of digital devices has led to a significant change in our lifestyle. In the current study, an unhealthy lifestyle in the form of prolonged use of electronic devices and lack of physical activity significantly contributed to adolescent obesity. A previous study conducted in Tabuk City revealed a significant association between the prolonged hours of watching TV per day and obesity among primary school boys aged nine to 12 years [31]. In Western Saudi Arabia, Al-Agha et al. [32] attributed obesity among children and adolescents aged two to 18 years to the increased time spent using electronic devices such as computers and video games for more than two hours per day, as well as a decreased time of practicing different types of physical activities for less than 30 minutes. A systematic review of factors contributing to obesity among children and adolescents in South Asia revealed a significant impact of prolonged TV watching, playing computer games, and lack of physical activities [33]. Another systematic review that included six longitudinal studies and one interventional study revealed a significant association between prolonged screen time and the incidence of obesity among children and adolescents [34]. The multivariable regression analysis in this study also revealed a significant contribution of physical inactivity to the development of obesity. Hence, it seems that children's prolonged engagement with digital technologies reduces their participation in outdoor activities, such as sports, exercise, parks, picnics, and camping. Moreover, children and adolescents' perception of safety concerns in their local neighborhoods contributes to increased passive entertainment [35]. Other studies have suggested that the use of devices is associated with the consumption of high-energy foods, such as snacks, sugary items, or drinks [36]. The consequences of increased screen time and decreased physical activity were observed during the COVID-19 pandemic as evidenced by the increased weight gain in children and adolescents compared with pre-pandemic rates [37].

The study found an association between a family history of obesity and adolescent obesity. Genetic predisposition may influence behaviors such as high food intake, physical inactivity, and decreased metabolism within families. Family members may also share similar unhealthy habits, creating an "obesogenic environment." The interplay between genetic predisposition and shared environmental factors may contribute to the increased risk of obesity among family members. Understanding the role of genetics can aid in risk stratification and personalized interventions for youth with a family history of obesity.

A noteworthy finding was that a substantial percentage of overweight or obese adolescents did not view obesity as a disease, which the study identified as a significant risk factor for obesity. Not viewing obesity as a disease may lead to a lack of motivation to make healthy lifestyle changes or seek treatment.

Overall, the findings highlight the complex interplay between genetic predisposition, environmental factors, and lifestyle choices in adolescent obesity. Effective prevention strategies should address these factors through a multi-faceted approach that includes school-based health education programs to promote healthy eating habits and address misconceptions, educating adolescents and their families about the health consequences of obesity, encouraging parents to support healthy lifestyles within the family environment, and providing opportunities for safe and engaging physical activity both inside and outside of school.

Limitations

This case-control study has the strength of identifying multiple risk factors simultaneously. However, its retrospective nature limits its ability to establish causation, and it is subject to selection bias. In addition, the study relies on self-reported data from questionnaires. Self-reported dietary intake and physical activity levels may be inaccurate due to recall bias or social desirability bias. These types of biases may reduce the power of the study results. In addition, the study was conducted in Alabnaa Schools in one Saudi city, i.e., Tabuk City. Therefore, the findings may not be generalizable to other populations with different cultural backgrounds or socioeconomic factors. Furthermore, the presence of confounding variables such as socioeconomic status or parental education level could influence both lifestyle factors and weight status.

Further studies are encouraged to use a multistage recruitment strategy and to stratify the sample based on relevant demographic variables (e.g., urban/rural location and socioeconomic status). Conducting the study in multiple schools in different regions of Saudi Arabia can capture a broader population demographic. These measures are proposed to significantly reduce bias. Statistical techniques, including multivariable regression analysis, can also account for the potential influence of confounding variables on the results.

## Conclusions

Certain factors increase the risk of overweight or obesity in adolescents, including frequent eating, prolonged use of electronic devices, family history of obesity, and the misconception that obesity is not an illness. These findings highlight the need for a health education campaign to raise awareness among students and parents about healthy lifestyles and eating habits. New school health programs need to be implemented to reduce passive sedentary time and the use of electronic devices and to promote physical activity among children. Additional intervention studies are needed to determine the impact of lifestyle and dietary changes on the incidence of adolescent obesity.

## Appendices

Questionnaire

1. Age (years) ----------------

2. Gender?

• Male

• Female

3. Height ---------------------

4. Weight -----------------------

5. How many meals do you take per day?

• Less than two

• 2-3

• 4-5

• More than 5

6. What is the type of the school kitchen?

• Junk foods

• Normal food

• Both

7. Do you drink sugar-sweetened beverages?

• Yes

• No

8. Do you drink carbonated drinks?

• Yes

• No

9. If yes, how many per day?

• One

• Two

• More than two

10. Do you engage in regular physical activity?

• Yes

• No

11. If yes, which type?

• Football

• Walking

• Cycling

• Other, specify -------------------------

12. Duration of your physical activity per week?

• 1 hour or less

• 2-3

• 4-5

• More than 5

13. Where do you have your activities?

• Home

• General gym

• Open place in your neighborhood

14. Do you receive advice about physical activities?

• Yes, usually

• Yes, but not usually

• Rarely

• No

15. How many scheduled physical activity sessions are in the school timetable?

• One

• Two

• Three

• More than three

16. Please tick the device you use daily.

• Playing device

• Mobile

• Laptop or iPad

• TV

17. What is the daily duration (hours) of using the above device?

• Two or less

• 3-5

• More than 5

18. What do you use your device for?

• Social communication

• News

• Education

• Playing or watching films

• Others, specify

19. Which way of recreation do you prefer?

• Physical activity

• Using your device

20. Do you eat while using your device?

21. How many hours did you sleep per day? -----------------------------

22. Do you believe that obesity is an illness?

• Yes

• No

23. Does your family have a history of obesity?

• Yes

• No

24. If you believe that you are obese do you want to lose weight?

• Yes

• No

25. If you believe that you are obese, did you try to lose your weight?

• Yes

• No
